# Modeling analysis of the correlation between duality innovation and multinational enterprise performance

**DOI:** 10.3389/fpsyg.2022.1000153

**Published:** 2022-10-18

**Authors:** Xinran Li, SzeTing Chen

**Affiliations:** Chinese International College, Dhurakij Pundit University, Bangkok, Thailand

**Keywords:** multinationals, corporate performance, corporate risks, innovation, correlation

## Abstract

In this study, we investigate how the influence of dual innovation affects the productivity of multinational enterprises (MNEs). Because of the rise of the knowledge-based economy, the capacity of multinational corporations (MNCs) to innovate technologically has become an increasingly important component in determining the extent to which they can compete in the global market. Models of Duality Innovation and Multinational Enterprise Performance with a Measurement of Corporate Risks from 2000 to 2015 were developed using corporate finance literature and data. The models show positive relationships between duality innovations and multinational enterprise performance. Furthermore, there has been an increasing level of corporate risks over the years when measured by both the duality innovation and multinational enterprise performance metrics. This article discusses the findings of this research project. It explains how they can help understand international enterprise performance while also explaining how to determine a potential risk profile for an individual or multiple companies. This knowledge is valuable because it helps us understand why some corporations succeed while others fail.

## Introduction

This paper aims to identify the relationships between Multinational Enterprise Performance and Duality Innovation. Duality Innovation is using both organic and acquisitive growth strategies by innovation to create a competitive advantage (Dong et al., 2021). This research attempts to discover if there are any associations between these two methodologies and their outcomes. This research project also aims to test for potential risks associated with standardized measurements for Multinational Enterprise Performance using data from 2000 to 2015 (Rugman et al., 2016; Gao et al., 2020).

To identify the relationships between Multinational Enterprise Performance and Duality Innovation. This paper explains how these measurements affect each other and why they might be linked together (Collings et al., 2019; Dong et al., 2021). Correlations between the metrics are positive, supporting the hypothesis that there is a connection between the two methodologies. Furthermore, risks associated with both of these metrics also have a positive correlation (Yan et al., 2021; Liu et al., 2022). This research supports existing literature that posits a direct relationship between corporate risk and business strategy (Rugman et al., 2016; Wu et al., 2019). The paper will be valuable to researchers because it will allow them to understand the relationship between the metrics and their impact on corporate performance. This paper aims to demonstrate that multinational corporations in the developed world should consider risks when making decisions about their business strategy. This research is relevant because it supports existing theory by showing a connection between the two metrics and how they are affected by corporate risks (Collings et al., 2019; Yi et al., 2020; Awan et al., 2022).

The significance of this study lies in the fact that it will provide light on the effect that the strategy has on the performance of MNEs, which is one of the most critical aspects of MNEs (Kyove et al., 2021). This article will demonstrate why risk analysis is necessary when utilizing data from various sources. It will also explain potential challenges associated with standardized assessments of the performance of multinational enterprises (Hussain et al., 2021). This study will be helpful to international organizations since it provides a new viewpoint on company strategy and risk assessment. Not only do multinational firms dominate the markets in which they operate locally, but they also confront intense competition on a global scale. Because they serve consumers in other nations, firms are forced to conform to those countries’ different demographics, legal needs, and cultural standards (Wu et al., 2019; Mustafa et al., 2022a). Therefore, they ought to consider how the differences between markets can influence the method they choose. This research is essential because it reveals how knowledge from studies like this may help multinational firms decide how to approach and compete in foreign marketplaces. This knowledge can be beneficial for multinational corporations.

The research is also relevant because only two studies (Yang et al., 2020; Mustafa et al., 2022b) have integrated the study of multinational enterprise performance with duality innovation. The first study uses data from 2014 to 2015 to identify which companies are more innovative and which are more exploitative. It focuses on identifying the companies that use both strategy measures and analyzing them to see if there is an association between duality innovation and corporate performance. The second study focuses on using Canadian Multinational Enterprise performance models (Li et al., 2018) and adds another year of analysis on a new set of data (Baig et al., 2022; Lu et al., 2022). Both studies show positive correlations between the two methodologies and their outcomes, but they do not explain why this might be. This research will support these studies by establishing a relationship between the two methodologies. Furthermore, this article will also define different risks that multinational corporations need to be aware of when making decisions about their business strategy. This paper will help multinational corporations make better decisions about performing in their foreign markets by identifying existing risks and demonstrating how they are linked to Duality Innovation.

The goal of this research is to investigate the links between the concepts of innovation and product innovation to answer the question, “Does innovation influence the effects of innovation?” The following examples demonstrate the importance of this research: To begin, it makes a substantial contribution to the study of a subject that, up until this point, has not received nearly as much focus as it should have: innovation in multinational organizations. Second, the influence technological advances have had on the prosperity of multinational corporations will be the paper’s topic in the next section. Thirdly, this research explores moderating factors that suggest potential avenues for formulating new policies.

The study begins by reviewing the relevant literature that integrates into the Multinational Enterprise Performance models. Then, a methodology for creating and integrating these models is discussed in more detail. Next, the results of this research project are given, along with an explanation for what they mean and why they are significant. Finally, potential risks associated with standardized measurements of Multinational Enterprise Performance are demonstrated. This paper concludes by discussing the implications and suggesting how these tools can be used to improve corporate performance. The literature review is divided into two main sections, the first being the literature review on Multinational Enterprise Performance and the second being the review of the relevant literature on Duality Innovation. By reading this paper, you will understand the research project and why it was carried out. The discussion consists of the development of the model itself, along with an explanation for all findings given in this research paper. This research will be valuable because it helps explain why some companies succeed in the long term.

## Literature review

Duality innovation is a methodology used by multinational corporations to determine how they can best export their products and services to foreign markets. This paper will use the Duality Innovation tool to identify how two or more types of innovation positively correlate to corporate performance. The other metric integrated into this study is Multinational Enterprise Performance (MEP). MEP measures a multinational’s potential for profit in each market it operates (Cooke et al., 2018; Lu et al., 2022; Mohsin et al., 2022b). It is an established metric created by the World Economic Forum and is used across many industries (Jamil et al., 2022; Wang et al., 2021). This study develops a multivariate method for combining the two measures to identify which companies are more innovative and which are more exploitative.

A pioneering model for measuring multinationals was developed in 1991 by the World Economic Forum as part of their Global Competitiveness Index (GCCI). Although this is an established metric, it does not approach MEP in terms of complexity. The GCCI includes an evaluation of national competitiveness (GCCI score), standards of living (GCCI income level), and social development (GCCI human development index). Although this is a valuable model for comparing global competitiveness, it does not consider the risks associated with implementing this methodology (Naseem et al., 2019; Yi et al., 2020; Christofi et al., 2021; Jin et al., 2021).

In Multinational Enterprise Performance, an NIE consists of three main elements: the number of countries the company has operations in (the size of the NIE), its market value (the scope), and the R&D it produces in each country (the degree). Therefore, the formula used to assess multinational performance is as follows: Where “N” represents the number of countries with which a firm has operations; “V” is market capitalization; “R&D” is research and development spending; and “EBITDA” is earnings before interest, taxes, depreciation, and amortization. When using this formula, multinational corporations must consider the risks associated with their foreign operations when making decisions about their strategy (Vahlne et al., 2018; Muhammad et al., 2019; Hsu et al., 2020; Ma et al., 2021).

### Main sources of risk

Before choosing a market in which to enter, multinational corporations are required to consider all of the future expenses carefully. Suppose your firm is thinking of creating a branch in South Korea, for example. In that case, you should consider the advantages and disadvantages of conducting business in that country and the prospect of facing competition from other global companies already established there (Zhu et al., 2018; Naiwen et al., 2021). Because the culture and policies of each nation are unique, multinational firms must adopt various strategies when entering new markets (Gul et al., 2021a,b). As a result, the degree of risk associated with various strategies for entering a foreign market varies significantly. According to Rashidin et al. (2020), it is recommended that multinational companies arrive at a conservative estimate of the absolute risk by taking into account all types of threats and basing their decisions regarding a country on the total amount, rather than relying solely on the principal component of the risk (i.e., government policy). When considering a direct investment, the threats most essential to consider are those related to the law, the environment, politics, the government, culture, and finances (Zheng et al., 2021; Xu et al., 2022). These threats are not unique to any one country and can potentially negatively impact economies worldwide (Li et al., 2021). Indeed, these factors must be considered before implementing any procedures. According to Zhu et al. (2017), once the operations have started, it is impossible to change these aspects immediately, but they can be handled with time and effort.

In addition to the main types of global risks, there are also risks associated with each country. These risks rely on factors that can vary from country to country, such as language, culture, economy, and infrastructure (Yang et al., 2020; Huang and Liu, 2021). When considering whether a country is a good market for its goods, companies look at the political environment, infrastructure quality, and competition from other multinationals (Rygh, 2020; Li et al., 2021). When doing business in other countries, multinational corporations must consider political risks. For example, if a company wants to do business in Iraq or Venezuela, it must first understand what this means for them operationally. This is a particular type of risk because the political environment can impact their operations, regardless of how it affects the rest of their business (Awan et al., 2021b; Cooke et al., 2018; Wang et al., 2018).

Multinational corporations must examine the reasons behind their decision to establish operations in nations with the unstable political climate. As mentioned earlier, the level of danger associated with traveling to any particular country can vary greatly depending on several different circumstances (Huang et al., 2021). These threats, their nature, and the degree to which they present themselves vary significantly from nation to nation (Meyer, 2018; Zhongjun et al., 2022). Companies assessing the benefits and drawbacks of entering a territory should consider the moves their competitors are making in the target market and their forecasts for the future of business in the region. In addition, they need to be aware of their individual risk preferences regarding the management method (Wu et al., 2020; Mohsin et al., 2021; Mustafa et al., 2022e). By having a clear picture of the risks they are taking on in advance, businesses can lessen the impact that the political hazards of conducting business in a foreign country have on their operations (Zhu et al., 2018; Khan et al., 2020; Mohsin et al., 2022a).

Cultural differences can pose a significant problem for multinational corporations entering new markets. Some countries may have barriers to cultural norms that are hard to overcome once implemented (Azam et al., 2022; Luiz and Barnard, 2022). Shirodkar and Shete (2021) notes that there are several ways for companies to deal with such barriers, including providing stakeholders with education in this area and building trust with the local community. To do so, companies should consult widely and seek information regarding the culture of the country they want to enter (Vahlne et al., 2018; Ahsan and Fernhaber, 2019; Zhou et al., 2022a). By doing so, they can identify these cultural barriers and create a plan to work around them. When entering a new market, corporations need to consider cultural differences, which can pose obstacles not just in the short term but over time as well (Zhang et al., 2019; Khan et al., 2020; Wu et al., 2020; Mustafa et al., 2022c).

Firms must also consider how well their business model will fit with the countries they want to operate in. For instance, if a company wants to do business in China, it should consider that Chinese culture is very different from that of the United States (Wrana and Diez, 2018; Papanastassiou et al., 2020; Mustafa et al., 2022d). Specifically, they must examine the differences between Chinese and American values and how these may impact operations. If a firm wants to do business in China but does not want to be affected by cultural differences between the two countries, it can simply avoid doing business there (van der Waal et al., 2021; Zhou et al., 2022b). However, if a company wants to run its businesses as usual but still move into this market, it must compensate for these differences and adjust its business model (Torrecillas and Brandão Fischer, 2021). Multinational corporations face many risks when setting up operations outside their home country (Feng and Wang, 2019; Narula and Pineli, 2019; Table 1 shows all the sources).

## Research data and methods

### Context of the study

The survey was conducted in 6 districts in Ho Chi Minh City: Tan Binh, Binh Thanh, Tan Phu, District 6, District 9, Thu Duc district, and Binh Chanh district. These districts were selected to ensure feasibility. In each district, the interviewer contacted the Tax Department to approach businesses. Sampling this way may not guarantee the rigorous standards of random sampling, but it is the best the authors can do given the limited time and budget.

### Data analysis

Primary data is cleaned, processed, and analyzed using descriptive and comparative statistical methods. In addition, the study uses the Binary logistic model to quantify the influence of the Government’s financial support policies on the decision to innovate enterprises’ technology. The model has a dependent variable, Y_i_ representing the decision to innovate technology, taking the value 0 if the firm HAS technological innovation and receiving the value one if the firm does NOT innovate. The independent variable (X_k_) includes the Government’s financial support policies (X_1_; gets the value 0 if the enterprise does not receive support; gets the value one if the enterprise receives the support); the Government’s technical support policies (X_2_; receive value 0 if the enterprise does not receive support; receive value one if the enterprise gets support); the operation period of the enterprise (X_3_; years); the size of the enterprise (X_4_; the number of employees), the profit of the enterprise is converted to the form of natural Logarithms (X_5_; thousand dongs); the enterprise’s debt is converted to the formation of natural Logarithms (X_6_; thousand dongs); the ratio of machine operating time (Xi_7_; % with i = $\bar{1,5}$ ; 1. Duration of fewer than 3 years; 2. From 3 to 5 years; 3. From 6 to 10 years; 4. From 11 to 20 years; 5. More than 20 years); interaction between the Government’s financial support policy and profit (X_8_).

General regression model:


(1) $$Ln\left(\frac{P_{i}}{1-P_{i}}\right)=\beta_{0}+\beta_{1}X_{1}+\beta_{2}X_{2}+\ldots +\beta_{k}X_{k}+u$$

With $\mathrm{Odds}=\frac{P_{i}}{1-P_{i.}}$

The regression model can be rewritten as:


(2) $$\mathrm{Ln}\left(\mathrm{Odds}\right)=\beta_{0}+\beta_{1}X_{1}+\beta_{2}X_{2}+\ldots +\beta_{k}X_{k}+u$$

Specifically, the model was applied to examine the influence of the Government’s financial support policy on the decision of technological innovation of SMEs in Ho Chi Minh City. Below are the variables (Table 1).

**Table 1:** Description of variables in the model.

Variable	Description	Expected sign	References
*Dependent variable*			
TI	Probability in the decision to innovate the technology of enterprises		
*Independent variable*			
SUPF	Enterprises received financial aid from the Government	+	Peltz and Weiss(1984), Zhou et al. (2014), Nugroho (2015)
*Control variable*			
SUPT	Enterprises received technical support from the Government	+	Peltz and Weiss (1984), Zhou et al. (2014), Sung (2019)
YR	Operation period of the enterprise	+	An and Danh (2015), Nhựt (2018)
SIZE	Size of the enterprise	+	Autry et al. (2010), Chang and Robin (2006), Correa et al. (2010), Gomez and Vargas (2009), Marom et al. (2019), Nhựt (2018), Zhou et al. (2014)
PROF	Profit of the enterprise	+	Loury (1979), Fudenberg and Tirole (1985), An and Danh (2015), Nhựt (2018)
LOAN	Loan of the enterprise	+/−	
UT_i_	The ratio of machine operating time	+/−	Siddharthan and Safarian (1997), Pandit and Siddharthan (1998)
*Intermediate variable*			
SUPF × LnPROF	Interaction between enterprises receiving financial aid from the Government and the logarithm of profits		

Compiled from previous studies of the authors.

## Research results and discussions

### Current status of technological innovation of SMEs in Ho Chi Minh City

According to the survey results of the research team, most of the enterprises in the research subjects are medium-sized enterprises, taking up 51.7%, followed by small-scale enterprises with 38.1%, and micro-enterprises account for 10.2%. The results from Table 2 show that the most significant difference between the researched enterprises is related to indicators such as the number of years in operation of the enterprise, the size of the enterprise, the profit and assets of the enterprise, particularly:

**Table 2:** Statistics of indicators with significant differences.

Indicator	Minimum	Maximum
Number of years in operation of the enterprise	4	58
Size of the enterprise	10	125
Profit of the enterprise	56	9,587,281
Assets of the enterprise	450	9,208,618

Study on SMEs in Ho Chi Minh City in 2019.

The difference in the above criteria reflects the level of technology and the process of improving and developing new products, which also varies among enterprises. Specifically, according to the author’s survey results, micro-enterprises that will enhance existing products account for 10.0%, while this percentage in small and medium enterprises is 19.4% and 28.7%, respectively. This shows that for medium-sized enterprises with a more significant number of employees, more considerable assets, and profits, the ability to apply technology to improve products will have more advantageous conditions. Moreover, the speed of technological innovation is higher, especially for medium-sized enterprises. Enterprises are constantly improving and developing new products to meet the increasingly competitive demands in domestic and foreign markets.

### The influence of the Government’s financial support policy on the ability of enterprises to innovate technology

In this section, the Binary logistic model will be used to quantify the effects of the Government’s support policies on a firm’s decision to innovate technology. The authors propose two research models: model 1 with factors affecting the decision to innovate the technology of enterprises; model 2: the interaction between the Government’s financial support policy and the profits of enterprises is measured to consider whether the remaining factors strongly influence the Government’s policy or not. Based on that, solutions are proposed to promote the effectiveness of the financial aid policy for the decision to innovate technology in SMEs in Ho Chi Minh City in particular and Vietnam in general (Tables 2 and 3).

**Table 3:** Logistic regression results.

Variable	Model 1 (without interaction variables)		Model 2 (with interaction variables)	
	Regression coefficient	Odds ratio	Regression coefficient	Odds ratio
SUPF	1.025434*	2.788307*	3.658999***	38.82247***
SUPT	1.914021***	6.780296***	1.836368***	6.273711***
YR	0.0238522	1.024139	0.0189981	1.01918
SIZE	0.0019905	1.001993	0.0000445	1.000044
UT1	−0.0078559	0.9921749	−0.0108889	0.9891702
UT 2	−0.032274	0.9682412	−0.0376392	0.9630604
UT3	−0.0460029	0.9550392	−0.0528057	0.9485643
UT4	−0.0473297	0.9537729	−0.0558349	0.9456952
lnPROF	0.1554975**	1.168239**	0.2242752*	1.251415**
LnLOAN	0.1330805*	1.142342*	0.1746729**	1.190857**
SUPF × LnProf			−0.2734846***	0.760724***
Constant	0.1536671	1.166103	0.0415198	1.042394

Results processed from Stataa 13, ***p < 0.01, **p < 0.05, *p < 0.1.

Regression results in both models show that the decision to innovate technology in enterprises is affected by factors other than the Government’s financial support policy, such as technical support policy, corporate profits, and debts. The results aligned with the research’s expectations (Mustafa et al., 2022c). The most substantial impact is the Government’s financial support, followed by technical support. However, when implementing the policy, it is also necessary to pay close attention to the profit factor of enterprises because this factor changes the effectiveness of the policy (Hu et al., 2021). Specifically, suppose the profit factor does not bind the Government’s aid for enterprises. In that case, the probability of a decision to innovate technology increases 2.78 times higher than that of enterprises that do not receive financial support (model 1). Other factors such as technical support from the Government, debts, and profits also positively affect the decision to innovate technology in enterprises (Mustafa et al., 2022c).

Considering model 2, the Government’s financial support is correlated with the profits of enterprises, bringing exciting results. Enterprises receiving financial aid from the Government have a higher probability of choosing to innovate technology up to 38.82 times compared to enterprises that do not (Zhao et al., 2021). However, governmental support for businesses with positive financial status and stable profits will not be adequate; as this form of support specifically reduces the probability of technological innovation of enterprises (the probability of technological innovation of enterprises receiving financial support from the Government is only 0.76 times that of enterprises that do not accept help). When receiving financial grants from the Government, enterprises focus on so-called technological innovations; their efficiencies are minimal (Awan et al., 2021a). The results also reveal the delay in policy implementation, causing the businesses that critically need the grant to seek other financial resources as they need immediate access to new machinery systems for their operations. Those financial resources might be loans from financial institutions, from existing profits of the business (the portion of profits that should be used for other activities). Thus, profitable firms will increase the probability of a decision to innovate by 1.25 times compared with good firms that do not innovate.

## Suggested solutions

Under the influence of the Government’s support policies on the decision to innovate the technology of SMEs, the following solutions are proposed:

Firstly, it is necessary to focus on restructuring the content, programs, and targeted enterprises that need financial support for the policy to be effectively promoted. The government’s financial support policies are suitable for each business, especially regarding the financial situation of enterprises.

Second, straitening tax and interest rate incentives for all fields related to technological innovation will create favorable and accessible conditions for businesses to access the government’s financial support policies. Through that, companies will have easier access to capital to actively invest in technological innovation, eventually improving labor productivity and competitiveness in the market.

Thirdly, it is crucial for businesses to proactively approach the government’s supportive policies and constantly improve their financial capacities through a wide range of channels to motivate the process of technology innovation actively. Furthermore, favorable conditions should be created to increase the competitiveness of enterprises and investments from external sources in the field of technology to expand production activities gradually and the scale of operations.

## Limitations and future research

This study does have certain limitations, even though it provides some helpful insights. To begin, even though a variety of classifications can be applied to the innovative performance of multinational corporations, the primary focus of this research was on product innovation. This was done so that survey data could be obtained regarding performance, similar to what was done in earlier studies concerning the manufacturing industry. Even if the creative performance criteria utilized in this method have been employed in previous studies, which lends credence to the research, the study may be deficient in other respects. The researchers who carried out this investigation made use of a generic survey instrument to classify the various forms of assistance provided by the government into three distinct categories. These categories were as follows: direct financial help, indirect aid in the form of innovation potential, and indirect assistance in the form of service in the distribution of the benefits of innovation. This question cannot be answered since there are much too many distinct kinds of aid programs. The results of future studies of innovation performance that study more subtle aspects of the service industry have the potential to provide more specific policy-related ramifications. As a consequence, there is a requirement for additional research that uses the information obtained from a questionnaire survey of individuals working for foreign firms.

## Conclusion

The decision to innovate technology in SMEs is influenced by many factors: the Government’s financial and technical support policies, the rate of machine operating time, assets, and profitability of the business, among which financial aid has the most decisive influence. However, for this policy to be effective, it is necessary to have a well-organized policy system designed to suit the needs of businesses and implemented in a more timely manner, especially the current financial situation of companies should be thoroughly examined before funding. Doing that will create a strong motivation for investment activities in technological innovation to help gradually; businesses expand their scale and improve their market competitiveness.

## Data availability statement

The raw data supporting the conclusions of this article will be made available by the authors, without undue reservation.

## Author contributions

All authors listed have made a substantial, direct, and intellectual contribution to the work and approved it for publication

## Conflict of interest

The authors declare that the research was conducted without any commercial or financial relationships construed as a potential conflict of interest.

## Publisher’s note

All claims expressed in this article are solely those of the authors and do not necessarily represent those of their affiliated organizations, or those of the publisher, the editors and the reviewers. Any product that may be evaluated in this article, or claim that may be made by its manufacturer, is not guaranteed or endorsed by the publisher.
